# Use of RNA Immunoprecipitation Method for Determining *Sinorhizobium meliloti* RNA*-*Hfq Protein Associations In Vivo

**DOI:** 10.1186/s12575-018-0075-8

**Published:** 2018-05-01

**Authors:** Mengsheng Gao, Anne Benge, Julia M. Mesa, Regina Javier, Feng-Xia Liu

**Affiliations:** 1 Soil and Water Science Department, Cancer and Genetics Research Complex, Room 330E, University of Florida-Institute of Food and Agricultural Sciences, Gainesville, 32610 USA; 2 Department of Plant Genetics and Breeding, China Agricultural University, Beijing, 100193 People’s Republic of China

**Keywords:** RNA immunoprecipitation, *S. meliloti* Hfq, Nodule lysate, Rhizobia symbiosis

## Abstract

**Background:**

Soil bacterium *Sinorhizobium meliloti* (*S. meliloti*) forms an endosymbiotic partnership with *Medicago truncatula* (*M. truncatula*) roots which results in root nodules. The bacteria live within root nodules where they function to fix atmospheric N_2_ and supply the host plant with reduced nitrogen. The bacterial RNA-binding protein Hfq (Hfq) is an important regulator for the effectiveness of the nitrogen fixation. RNA immunoprecipitation (RIP) method is a powerful method for detecting the association of Hfq protein with specific RNA in cultured bacteria, yet a RIP method for bacteria living in root nodules remains to be described.

**Results:**

A modified *S. meliloti* gene encoding a His-tagged Hfq protein (Hfq^His^) was placed under the regulation of the native Hfq gene promoter (P_*hfq*sm_). The *trans* produced Hfq^His^ protein was accumulated at its nature levels during all stages of the symbiosis, allowing RNAs that associated with the given protein to be immunoprecipitated with the anti-His antibody against the protein from root nodule lysates. RNAs that associated with the protein were selectively enriched in the immunoprecipitated sample. The RNAs were recovered by a simple method using heat and subsequently analyzed by RT-PCR. The nature of PCR products was determined by DNA sequencing. Hfq association with specific RNAs can be analyzed at different conditions (e. g. young or older root nodules) and/or in wild-type versus mutant strains.

**Conclusions:**

This article describes the RIP method for determining *Sinorhizobium meliloti* RNA*-*Hfq associations in vivo*.* It is also applicable to other rhizobia living in planta, although some tissue-specific modification related to sample disruption and homogenization may be needed.

## Background

*Sinorhizobium meliloti* (*S. meliloti*) forms an important endosymbiotic partnership with *Medicago truncatula* (*M. truncatula*) roots which results in the development of specialized organs called root nodules [1]. Bacteria live within root nodules where they function to fix atmospheric N_2_ and supply the host plant with reduced nitrogen. This interaction of *S. meliloti* and *M. truncatula* provides a model system to study the molecular basis of *Rhizobium*-legume N_2_-fixing symbioses [2, 3]. Forty to sixty (40 – 60) million tons of nitrogen are fixed annually by the *Rhizobia*-legume N_2_-fixing symbioses in cultivated legumes, saving about $10 billion on nitrogen fertilizer [4, 5]. *S. meliloti* RNA-binding protein Hfq is an important regulator that governs the effectiveness of the *S. meliloti*-legume interaction [6–8]. The N_2_-fixing efficiency is severely reduced if *S. meliloti* mutants carry mutations in Hfq gene (*hfq*) and this reduced efficiency is accompanied by reduced stress tolerance.

Hfq fulfills its function through association with specific RNA sequences [9]. Putative Hfq-binding sites i.e., free 3′-hydroxyl end of an oligo-U stretch or A/U-rich regions in *S. meliloti* RNA molecules are predicted in silico [10]. Two lines of experimental evidence, with a strong genetic base, have now demonstrated that Hfq binds to A/U-rich regions in mRNAs for both ExpR and for FixL proteins in the bacterium and that the bindings cause changes the stability and translation efficiency of those RNAs [11, 12]. A global regulatory role for Hfq in controlling gene regulation in the bacterium is supported by high-throughput transcriptomic studies, which demonstrated that, dependent on conditions, Hfq can regulate large number (1315) of *S. meliloti* RNAs including noncoding regulatory small RNAs [13].

Several RIP methods have been used for detecting the association of Hfq with specific RNA [11, 13–17] in cultured bacteria. Although very fruitful to identify and validate the Hfq-RNA association in bacteria grown in free-living state, these methods are unable to accurately detect symbiosis Hfq-RNA association because many symbiosis genes mainly express inside the host tissue [18]. Furthermore, these methods require many bacterial cells which limit their usefulness in root nodules where bacteria persist in a relative small number due to a control imposed by host plants [19]. In addition, most methods developed to recover immunoprecipitated RNA from samples involve multistep extraction using phenol-chloroform extraction and elution procedures. While generally effective, these methods are time consuming and create the potential for RNA loss during each processing step as already discussed by other investigators [20]. However, a RIP procedure for identifying Hfq associated RNA (hereafter Hfq RNA) from root nodule lysates remains to be described.

**Fig. 1:**
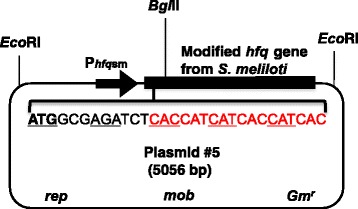
Structure of the plasmid p#5. p#5 contains the modified Hfq gene from *S. meliloti* with sequence (red) for an affinity His-tag to produce Hfq^His^. The gene is cloned under the regulation of the native Hfq gene promoter (P_*hfq*sm_) [37]. p#5, a derivative of pBBR1MCS-5 [38], also carries a gentamicin resistance gene (Gm^r^), the broad host range replication (*rep*) origin and the sequence (*mob*) allowing for conjugal mobilization

Previously, we used a RIP procedure to identify Hfq RNA from cultured bacteria [11]. For that RIP experiment, we constructed a broad host range *trans*-Hfq^His^ production plasmid called p#5 [11] (Fig. 1). The *trans* produced His-tagged Hfq (Hfq^His^) is accumulated at their nature levels during different stages of culture growth, allowing Hfq RNA to be isolated in different growth conditions.

During that work, we realized that the use of the p#5 based RIP procedure could be further extended to assist the rhizobia research for determining *S. meliloti* RNA*-*Hfq associations in root nodules if the p#5 is stable in plants and complements Hfq mutants for symbiosis. This idea was initially tested in young root nodules, which revealed *fixLJ* mRNA as a Hfq RNA [12]. Here, we continue testing the idea and demonstrate that p#5 is stable in plants and complements Hfq mutants for symbiosis. Testing the procedure in matured nodules (49 days post-inoculation) revealed smelA075 as a Hfq RNA. This newly characterized regulatory RNA is conserved in rhizobia and has been proposed to play a role in stress tolerance during the symbiosis [21].

## Methods

### Reagents, Materials and Equipment

Nuclease-Free Water, DNA-free Kit, and Phosphate-Buffered Saline (PBS) were purchased from Ambion (TX, USA). Cell Extraction Buffer, anti-His were purchased from ThermoFisher Scientific (CA, USA). The SuperScript VILO cDNA Synthesis Kit, Bacto-tryptone Yeast extract and CaCl_2_ were purchased from Fisher Scientific (NJ, USA). Daynabeads Protein G was purchased from Life technologies (CA, USA). Power SYBR Green PCR Master Mix was purchased from Applied Biosystems (CA, USA). Sonic Dismembrator Model 100 was purchased from Fisher Scientific (CA, USA). ORBIT 1900 was purchased from Labnet International, Inc. (NJ, USA). 18 l Freeze Dry System (LABCONCO) was purchased from Labconco Corporation (MO, USA).

### Plant Growth and Inoculation

Cultures of the *S. meliloti* 1021 [22], Hfq mutant [23], and Hfq^His^ production plasmid /mutant [11] strains were grown to mid-log phase in tryptone yeast (TY) medium [5 g/l Bacto-tryptone, 3 g/l Yeast extract, 6 mM CaCl_2_ (added after autoclaving), pH = 7.2] [24] and centrifuged. The bacterial pellets were resuspended in an equal volume of water for inoculation onto seedlings. Seeds of *M. truncatula* A17 from the South Australian Research and Development Institute were surface sterilized with 95% ethanol and 6% hypochlorite, followed by extensive washing with sterile water. The seeds were kept at 4 °C overnight and then transferred to water agar plates. When seedling roots were 1.5 to 2 cm long, the seedlings were transferred to seedling growth pouches (Mega International, MN, USA), which were wetted to saturation with 9 ml of sterile, fourfold-diluted, and N-free Jensen’s medium [CaHPO_4_ 0.1%, K_2_HPO_4_, MgSO_4_ and NaCl each 0.02%, FeCl_2_ 0.01%] [25]. There were four holes, about 1 cm each, made in the punched bottom of the seed trench with sterile forceps and a seedling was carefully inserted through each hole with the root oriented toward the bottom and the cotyledon in the trench. Pouches were kept in an upright position in a box with spacers between sets of 10 to 15 pouches to prevent bending, incubated in a growth chamber (at 24 °C under a cycle of 16 h of light and 8 h of darkness), and restored back to original moisture levels each day with sterile water. When seedling main roots were 7 to 10 cm long (usually after 4 days of growth), seedlings were inoculated along the length of the root with 100 μl of bacterial suspension per seedling. The nodules on the primary root were harvested from plants on 10-, 18-, and 49- days post-inoculation (dpi).

### Measuring Shoot Dry Mass

Remove shoots from roots by cutting. Dry shoots in a freeze dryer Labconco 96 h. Let the shoots warm in a dry environment (a Ziploc bag will keep moisture out). Once the shoots have warmed weigh them on a scale (APX-60, d = 0.1 mg, Denver Instrument, Bohemia, NY). Average value of shoot dry mass for bacteria infected plants that differ significantly from the corresponding value for plants inoculated with the rhizobia-free water according to Student’s t test are indicated as follows: *, *P* < 0.001; **, *P* < 0.005.

### Reverse Transcription (RT)

RNA obtained by heat release were used in RT-PCR mixtures as described [11] with modifications. Thin-walled RT-PCR tubes were used, the reaction volume was scaled down to 10 μl, and reactions were run in a PTC-1148 thermal cycler with a hot bonnet (Bio-Rad Lab., Inc., Hercules, California). Following 10 min of incubation at 25 °C, the cDNA was synthesized at 50 °C for 90 min and heat denaturation of the enzyme at 85 °C for 5 min and hold at 4 °C.

### Quantitative PCR (qPCR)

cDNA samples were analysis by qPCR as previously described [12] with primers listed in Table 1. Briefly, 16S rRNA gene was used as internal control because cycle threshold (Ct) values of this gene is similar under several conditions [26]. A qPCR mix contained: 5 μl of SYBR Green Master mix, 1 μl of 5 μM stock solution for each primer, 20 ng of cDNA, and a proper amount of water to bring the total volume to 10 μl. qPCR reactions were run on a StepOnePlus real-time PCR system (Applied Biosystem, Fisher Scientific, NJ, USA). Reaction conditions were: 40 cycles at 95 °C for 15 s, 60 °C for 60 s, and 72 °C for 60 s, followed by a melt curve. RNA samples containing no RT were run as controls to ensure that samples were free from DNA contamination. Melt curve tests did not exhibit a second melting temperature for primer pairs used. qPCR data were processed using StepOne software version 2.2.2.

**Table 1:** RT-PCR Primers

	Target	Primer name	Left primer sequence (5′-3′)	Primer name	Right primer sequence (5′-3′)	Reference
1	Smrc7	MG3462	GCACTCATACAATGCCGTGA	MG3463	CTCTTTGAAAGCGGGACAAA	This work
2	SMrc15	MG2290	GGTGCATCTAGCGGCTTTCT	MG2291	GGGCCCTTTCAGTTGTGAAG	[11]
3	SMrc16	MG2292	CCACCGCAGCAGCTGTT	MG2293	GGCCCTTGTAGTTGTGAAGGTA	[11]
4	SMrc45	MG2294	TCGATTAGGTGAGGTTATCG	MG2295	CGGTTGGCCGGAATAGC	[11]
5	rRNA-16S	AB2777	GATAAGCCGAGAGGAAGGTG	AB2778	GTGTAGCCCAGCCCGTAAG	[40]
6	smelA075	RJ11	GTCGAAGCGCTGTACCT	RJ16	GGGAGGAGGTGGCTCGGGG	This work
7	Intergenic region between Smc00849-Smc00850	In850F	ATTTCTTCAATGACGTTCTCGTCA	In849R	ATACGTTCAAATTTTATCAT	[31]

## Results

### Hfq^His^ production Complements Hfq Gene Mutations for the Symbiosis

**Fig. 2:**
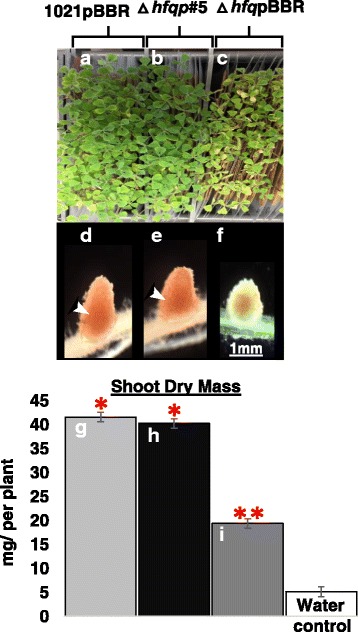
Symbiotic phenotypes of *S. meliloti* 1021Δhfqp#5. Leaves of plants inoculated with *S. meliloti* strains **a**, Rm1021pBBR1MCS-5; **b**, 1021∆hfqp#5; and **c**, 1021∆hfq pBBR1MCS-5. **d**, **e** and **f**, Corresponding representative nodules. Arrowheads indicate legHb protein produced in nitrogen fixation zone. **g**, **h**, and **i**, Shoot dry mass mean values of 2 independent cultures are given in mg/plant ± SEM. At least 56 plants were used per culture for each independent culture (*n* = 56). Average values of shoot dry mass for bacteria infected plants that differ significantly from the corresponding value for plants inoculated with water were determined using Student’s t test and indicated as follows: *, *P* < 0.001; **, *P* < 0.005

As previously reported [2, 27], and as shown in Fig. 2, inoculation of *M. truncatula* roots with wild-type *S. meliloti* induced cylinder-shaped N_2_ fixing root nodules, which maintained their morphology and function for a period of 49 days following inoculation (Fig. 2d). The nitrogen fixation rescued nitrogen starvation of *M. truncatula* plants as evidenced by their green-colored leaves (Fig. 2a) and normal shoot dry mass (42.2 ± 0.9 mg per plant, *n* = 56) (Fig. 2g). By comparison, inoculation of *M. truncatula* roots with Hfq mutant bacteria carrying p#5, either *S. meliloti* 8530∆hfq (*n* = 34, data not shown) or *S. meliloti* 1021∆hfq, also induced cylinder-shaped nitrogen fixing root nodules (Fig. 2e). The plants had green-colored leaves (Fig. 2b) and their shoot dry mass was normal (40.3 ± 0.6 mg per plant, *n* = 56) (Fig. 2h). The green leaves and normal shoot mass reflected the normal nitrogen fixation carried out by wild-type *S. meliloti*. Inoculation of *M. truncatula* roots with the Hfq mutant bacteria induced small white nodules (Fig. 2f)**.** The plants had yellowish leaves (Fig. 2c) and reduced shoot mass (20.1 ± 0.6 mg per plant, *n* = 56) which were an indication of nitrogen starvation (Fig. 2i). There were no visible changes on *M. truncatula* roots that were inoculated with rhizobia-free water (data not shown). The similarities between functions of *M. truncatula* nodules formed by the Hfq mutant strain carrying p#5 plasmid and by wild-type strain were further compared for p#5 restored nodulin LegHb (leghemoglobin, defined as pink-colored nodules, is required for nitrogen-fixation [28]). In normal *M. truncatula* nodules, LegHb production is accumulated in the nodules containing nitrogen fixing bacteria as indicated by arrowheads in Fig. 2d and e. The production of LegHb was observed both in nodules formed by wild-type *S. meliloti* (Fig. 2d) and by Hfq mutants carrying p#5 plasmid (Fig. 2e), but much less in nodules formed by the Hfq mutant (Fig. 2f). Our results demonstrated that *S. meliloti* Hfq mutant strains carrying the modified Hfq gene complemented Hfq gene mutations for nodule morphology, plant nodulin LegHb production, and the nitrogen fixation.

### The Hfq^His^ Production Plasmid P#5 Is Stably Maintained in *S. meliloti*

The gentamicin is the antibiotic used to select for maintenance of the broad-host-range Hfq^His^ production plasmid p#5, but it is toxic to plants. We had to inoculate plants with bacteria in the absence of the selection for plasmid maintenance. To assess plasmid stability in planta, we crushed nodules and plated nodule bacteria on nonselective media and subsequently screened for the plasmid marker gentamicin (Gm^r^) by replica plating on the agar containing the antibiotic at the concentration of 50 μg/ml. At the end of the experiment (49-dpi), about 85% of the Hfq mutant bacterial cells retained the p#5 plasmid and only 65% of them retained the parental plasmid pBBR, presumably because of Hfq regulated stress resistance functions [6, 7, 13] which are essential for survival of Hfq mutant bacteria in root nodules.

### The P#5 RNA Immunoprecipitation (RIP) Method

**Fig. 3:**
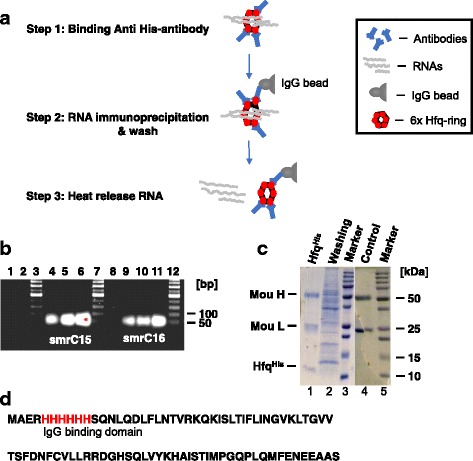
Testing the p#5 method. **a** An overview of the method. Hfq forms a hexametric ring for action [37] **b** smrC15 (lanes, 4, 5, and 6) and smrC16 (lanes 9, 10, and 11) RNAs were detected by RT-PCR in the samples heat-released from Hfq^His^, but not in the negative control samples (lanes 1 and 2). PCR failed to amplify genomic DNA in RNA samples (lane 8) with primers In850F and In849R (Table 1, [31]). **c** An image of 12% SDS-PAGE gel stained with 1% Coomassie blue R-250. Lane 1: The immunoprecipitated Hfq^His^ from p#5 nodule samples. H and L are heavy and light chains of the mouse anti-His antibody, respectively. Lane 2: Protein contents in the ‘first wash’ of the p#5 nodule sample. Lane 3 and 5: Protein molecular markers to calculate sample molecular weights. Lane 4: Precipitated antibody from the negative control. **d** The sequence and the position of the His-tag

We tested the p#5 RIP method by targeting SmrC15 and SmrC16, two previously identified Hfq RNAs of moderate abundance in nodule-associated bacteria [29, 30]. In this test, nodules formed by *S. meliloti* Hfq mutant bacteria carrying p#5 (named Sm1021∆hfqp#5, [12]) were harvested on the 49-dpi and ground to very thin powder in liquid nitrogen in a mortar with a pestle, waited for liquid nitrogen to evaporate. Then we immediately transferred an aliquot of nodule powder (100 mg) into 1 ml of TRIZOL for total RNA isolation and another aliquot (2 g) for RIP. The latter (2 g of nodule powder) was processed as follows: The sample was suspended in pre-chilled Cell Extraction Buffer (50% [wt/vol] suspension), incubated for 3 min on ice, sonicated with Sonic Dismembrator Model 100 five times (15 s per cycle at 28-30 w [11]). Resulting lysates were incubated at 4 °C for 10 min for homogenization. Cell debris in the lysates were pelleted by centrifugation for 20 min at 4000 x g at 4 °C and then the pellet was discarded. Six-hundred-microliters (600 μl) of cleared lysates were processed by the p#5 RIP method shown in Fig. 3a. To obtain efficient binding anti-His antibody (see Step 1), the cleared lysates containing Hfq^His^-RNA molecules were first diluted (4:1 [wt/vol]) in PBS (pH = 7.0) to reduce viscosity and then mixed with 10 μg of anti-His antibody. The mixture was incubated on a rotator at 4 °C for 30 min to allow the binding to occur [11, 14], and the resulting material was immunoprecipitated on 1.5 mg IgG beads (see Step 2) by consecutively incubating aliquots of the material with the beads for 10 min at room temperature. Then those beads were washed five times with PBS. Fifty percent (50%) of the bead slurry was frozen for sodium dodecyl sulfate-polyacrylamide gel electrophoresis (SDS-PAGE) analysis after the second wash. The remaining beads were suspended in 20 μl of Ambion nuclease-free water, then diluted (5:1, 1:1 and 1:5 [vol/vol]) for a thorough suspension of beads and for reducing potential inhibitors presented in processed samples therefore to reduce and prevent false-negative results. Samples were heated to 90 °C for 30 s to release RNA [11, 20] (see Step 3), chilled on ice for 2 min, and assayed immediately for RNA by RT-PCR. The amount of total RNA in RT-PCR mix was determined by Bioanalyzer data. A control experiment using lysates of nodules formed by wild-type bacterium (*S. meliloti* 1021 in which Hfq was not tagged with hexahistidine) was processed in parallel as described before [12, 15]. The oligonucleotide primers used for the RT-PCR were in Table 1**.** Genomic DNA specific primers In850 and In849 [31] (Table 1) failed to amplify genomic DNA in RNA samples which indicated that genomic DNA in RNA samples was below the level detectable by PCR (Fig. 3b, lane 8). RNA specific primers amplified SmrC15 cDNA (85 nt) (Fig. 3b, lanes 4, 5, and 6) and SmrC16 cDNA (60 nt) (Fig. 3b, lanes 9, 10, and 11) in all the diluted, heat release RNA content of p#5 nodule lysate. Because the amplification worked best for the smallest dilutions [5:1] in both cases of SmrC15 (lane 6) and SmrC16 (lane 11), there were no obvious inhibitions to be reported in those samples. DNA sequencing confirmed the PCR amplified bands (Fig. 3b, lanes 4, 5, and 6) as SmrC15 cDNA and the segment of confirmed sequence was as follow: CCTCCCCAGCCGCTGCAGCAGCTGTT. Also, DNA sequencing confirmed the PCR amplified bands (Fig. 3b, lanes 9, 10, and 11) as Smrc16 cDNA and the segment of the confirmed sequence was as follow: CCTCCCCAGCCGCTGCAGCAGCTGTT. Primers failed to amplify either SmrC15 or SmrC16 cDNA from control sample nodule lysates (Fig. 3b, lanes 1 and 2) which indicated that the His-tag worked effectively. Furthermore, our results were consistent with GUS gene fusions experiment data which showed the accumulation of SmrC15 is stronger than SmrC16 in planta [32]. Taken together, we concluded the followings: First, Hfq^His^ protein bound RNA specifically in *M. truncatula* nodules. Second, the p#5 RIP method worked properly. We routinely recovered sufficient amounts of Hfq^His^─anti-His antibody complex from 100 μl of frozen bead slurry to detect Hfq^His^ band by Coomassie staining (Fig. 3c). The clean protein band of Hfq^His^ with size about ~ 11 kDa was further verified by LC-MS (Liquid Chromatography – Mass Spectrometry) analysis, confirming the His-tag at the N-terminus of Hfq^His^ (Fig. 3d).

### Using the P#5 RIP Method Discovers New Hfq RNA smelA075

To test the generality of the p#5 RIP method, we targeted the *S. meliloti* smelA075 [13]. The smelA075 is a stress-induced small regulatory RNA with an uncharacterized relationship with Hfq, although it was seen in 5-week-old *M. truncatula* nodules [33] and exhibited Hfq responsive accumulation in cells grown in TY medium [23].

**Fig. 4:**
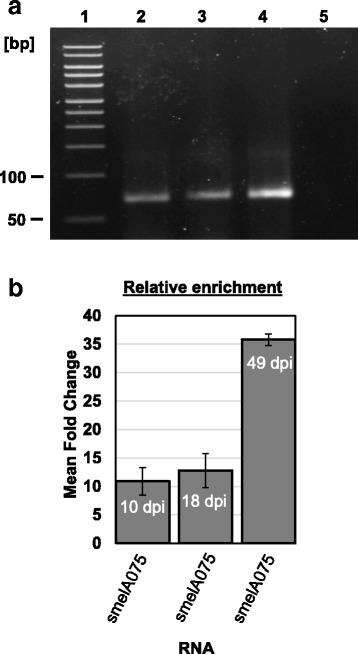
Hfq complexes smelA075 in *M. truncatula* nodules. **a** RT-PCR detected smelA075 from Hfq complexes immunoprecipitated from lysates of *M. truncatula* nodules 10-dpi (lane 2), 18-dpi (lane 3) and 49-dpi (lanes 4), but failed to detect smelA075 from control samples (lane 5). Markers (lane 1). This experiment was repeated twice and similar results were obtained (data not shown). **b** smelA075 immunoprecipitated with the Hfq^His^ was analyzed by qPCR. Relative enrichments were calculated with the 2^-∆∆Ct^ method [39]. The data are presented as mean fold change ± SEM and normalized to *S. meliloti* 16S rRNA gene

The smelA075 RNA was immunoprecipitated specifically with the p#5 procedure from 300 mg of nodule lysate at different nodule stages (10-, 18- and 49- dpi). RT-PCR data showed (Fig. 4a, lanes 2, 3, 4) the existence of smelA075 in RNA-Hfq complexes. Two primers used in PCR experiment, were RJ11 and RJ16 (Table 1). DNA sequencing confirmed the PCR amplified band (Fig. 4a, lane 2) as smelA075 cDNA and the segment of the confirmed sequence was as follow: CCTCCCACGGCGCCCGGCATTCGGT. The primers failed to amplify smelA075 from the control experiment as shown (Fig. 4a, lane 5)**.** Quantitative PCR analysis (Fig. 4b) further indicated that the relative enrichment of smelA075 by the protein was nearly tripled in matured nodules (49-dpi compared to 18-dpi, Fig. 4b) which indicated a tightened regulation of smelA075 by Hfq at the later stages of the symbiosis.

The fact that small RNA smelA075 shows a broad distribution pattern within the Rhizobiales [21] and carries three anti-Shine Dalgarno (anti-SD) sequences makes smelA075 likely to involve in targeting multiple downstream mRNAs.

## Discussion

Specialized RIP methods are required to analyze RNA-protein association during the N_2_-fixing symbiosis for two main reasons: First, symbiosis RNAs are often nodule-specific. Second, the number of bacteria in root nodules are relatively small compared to the bacteria grown in free-living state due to the host plant control [19]. Those make it difficult to use an in vitro RIP method for an in vivo study. There are very few protocols that work in N_2_-fixing symbiosis to produce rapid, direct information on RNA-Hfq association, except one [12] that was only tested in early stages of N_2_-fixation. We now describe a simple RIP method for determining *S. meliloti* RNA-Hfq association in all stages of N_2_-fixing symbiosis. The method has the necessary combination of simple procedure, sensitivity and consistent results to be a useful tool for determining *S. meliloti* RNA*-*Hfq associations in vivo*.*

In this in vivo RIP method, 2 g of nodule material can be processed in a short period of time without using phenol extraction. This method permits to reduce potential Hfq RNA lose during the extraction steps. The *trans*- produced Hfq^His^ protein has a sensitivity for recovering Hfq RNA of moderate abundance (Fig. 3b) and low abundance transcripts under optimal conditions [12]. Heat release was first used in recovery viral RNA from complex stool sample as described by Schwab et al. [20]. We modified Schwab’s method by shortening the time of heat from 5 min to 30 s (at 90 °C) to avoid RNA degradation. We used genome specific primers to detect trace DNA contamination for reducing the risk of false-positive results. We used dilution to reduce potential inhibitors presented in processed samples therefore to reduce false-negative results. DNase1 can be used to remove DNA contamination if necessary. The SuperScript VILO enzyme has optimal reaction temperature of 50-55 °C and can be used in reverse transcription for difficult templates such as small RNAs with secondary structures.

Although this procedure is designed for small scale analysis of the immunoprecipitated RNA from plant and the identity and relative amount of RNA sequence in control and immunoprecipitated samples are determined individually by RT-PCR and by quantitative RT-PCR, the amount of starting material can be scaled up and the RIP method can theoretically be combined with microarray technology or RNA sequencing to identify immunoprecipitated RNAs on a “genome”-wide basis. In fact, high-throughput RIP-chip and RIP-Seq methods have already been reported [34, 35].

## Conclusion

This article describes the RIP method for cells of the model symbiotic bacterium, *S. meliloti.* Hfq is conserved among the nodule forming symbiotic bacteria [36]. Therefore, the p#5 RIP method will have broader applications in study RNA*-*Hfq associations of *Rhizobia*-legume symbioses (e. g., *Azorhizobium*, *R. leguminosarum*). Some tissue-specific modifications related to sample disruption and homogenization may be needed.

## Abbreviations

dpi: Days of Post-Inoculation
Hfq RNA: RNAs that Associate with Hfq
Hfq^His^: His-tagged Hfq Protein
LC-MS: Liquid Chromatography – Mass Spectrometry
LegHb: Leghemoglobin
PCR: Polymerase Chain Reaction
qPCR: Quantitative PCR
RIP: RNA Immunoprecipitation
RT-PCR: Reverse Transcription-PCR
SDS-PAGE: Sodium Dodecyl Sulfate-polyacrylamide Gel Electrophoresis
SEM: Standard Error of the Mean
TY: Tryptone Yeast Medium
